# Designing for generalism, courage, and systems: a curriculum scholarship account of an integrated family medicine and primary care block

**DOI:** 10.3389/fmed.2026.1870368

**Published:** 2026-08-05

**Authors:** Baraa Alghalyini, Abdul Rehman Zia Zaidi, Racha Kamal Khaled, Mohammad Ameen Alswes, Helen P. Batty

**Affiliations:** 1 Department of Family and Community Medicine, College of Medicine, Alfaisal University, Riyadh, Saudi Arabia; 2 Department of Family and Community Medicine, Temerty Faculty of Medicine, University of Toronto, Toronto, ON, Canada; 3 School of Kinesiology and Health Science, York University, Toronto, ON, Canada; 4 Department of Family Medicine, King Faisal Specialist Hospital and Research Center, Riyadh, Saudi Arabia

**Keywords:** academic partnerships, clinical uncertainty, curriculum design, experiential learning, family medicine, medical education, Primary Health Care, systems thinking

## Abstract

**Background:**

Pre-clerkship Family Medicine teaching often covers content well but does too little with experiential immersion, structured reflection, and the open engagement with clinical uncertainty that defines generalist work in practice. We describe the partnership-based redesign of the COM353 Family Medicine and Primary Care block at Alfaisal University, Riyadh, with student-reported outcomes from the first delivered cohort. The paper is offered as a worked account of how academic-academic and academic-service partnerships can themselves become curriculum.

**Pedagogical framework:**

The redesign rests on three commitments developed in dialog with the Department of Family and Community Medicine at the University of Toronto: generalism as a clinical method that has to be taught deliberately, clinical courage as a disposition that can be cultivated, and community-based practice as a primary site of clinical learning. Teaching is built around an iterative concept-experience-reflection cycle, with didactic sessions, structured Primary Health Care Center placements, simulation, problem-based learning, and reflective writing.

**Learning environment and pedagogical format:**

COM353 is a four-week mandatory block for third-year medical students delivered through formal partnerships with Primary Health Care Centers in Riyadh and aligned with Saudi Arabia’s Vision 2030 health workforce priorities. Learning objectives target seven domains spanning generalist competencies, experiential learning, uncertainty tolerance, reflection, and systems thinking.

**Preliminary results from the first delivered cohort:**

A post-block survey of the January 2026 cohort produced 96 completed responses (response rate 76.2%). Domain means ranged from 3.90 to 4.27 on a five-point Likert scale, with Cohen’s d 0.92 to 1.58. Multiple linear regression identified Systems Thinking (*β* = 0.727), Reflection (*β* = 0.295), and Learning Environment (*β* = 0.293) as significant predictors of overall block satisfaction, in a model explaining 78.2% of the variance.

**Discussion and implications:**

A pre-clerkship block built on explicit pedagogy and run through partnership was associated with coherent, favorable student-reported outcomes across the competencies it set out to develop. That systems thinking emerged as the dominant predictor matters for design: when students grasp healthcare as a system, the rest of the curriculum lands harder. The model has practical relevance for medical schools in Gulf and other LMIC settings working toward PHC-centered health system reform.

## 1 Introduction

Health systems worldwide remain under pressure from aging populations, rising non-communicable disease, and widening inequities. The Astana Declaration reaffirmed what Alma-Ata argued about four decades earlier, that Primary Health Care is the foundation of equitable health systems (1). We use Primary Health Care in the sense set out by the World Health Organization, as a whole-of-society approach with three components: integrated health services with primary care at their core, multisectoral policy and action, and empowered people and communities (2). Primary care is the first-contact, continuous, comprehensive, and coordinated clinical service that family medicine delivers, and it is the component of Primary Health Care that an undergraduate block can most directly develop. International bodies continue to call for a strengthened primary care workforce. Medical schools have been slow to answer.

The problem is structural. Most undergraduate curricula remain organized around hospitals and oriented toward specialty practice, with clinical training gravitating to tertiary wards where presentations are filtered and the physician’s role is narrowly procedural. By the time many students encounter primary care in any sustained way, their professional identities have already begun to settle around images of medicine that exclude the longitudinal, the preventive, and the community-based. Parekh and colleagues documented this dynamic in reflective diaries from UK medical students, where the hidden curriculum systematically devalued general practice through absent role models, dismissive framings, and the linking of professional prestige to procedural specialties (3). The pattern is not confined to one country.

Two pedagogical priorities deserve attention in any pre-clerkship block aiming to take Primary Care seriously. The first is uncertainty. Family physicians routinely make decisions with incomplete information and manage undifferentiated presentations, yet medical students are usually taught to resolve uncertainty rather than work within it. Tolerance of uncertainty is not a fixed trait but a capacity that can be developed through exposure, modeling, and reflective engagement (4, 5). The concept of clinical courage, drawn from Reis-Dennis and colleagues’ normative analysis of corrective virtues, captures what students need: not recklessness, but the discipline to act ethically when certainty is unavailable (6).

The second priority is systems thinking. Physicians who understand how healthcare is organized, who coordinates care, how referral pathways function, and where resources concentrate or thin out are better positioned to serve patients and advocate for communities. Gonzalo and colleagues have argued for Health Systems Science as a third pillar of medical education alongside basic and clinical science, with systems thinking serving as the integrative domain that connects patient safety, quality improvement, population health, and interprofessional collaboration into coherent professional practice (7, 8).

These priorities require institutional structures: partnerships between academic departments and PHC delivery sites, alignment between educational objectives and health system priorities, and the deliberate framing of community-based settings as legitimate learning environments. In the Gulf region, this carries particular urgency. Saudi Arabia’s Vision 2030 transformation positions the family physician as central to universal health coverage, yet the educational infrastructure to produce that workforce at scale remains under-developed (9). Academic institutions that orient their curricula toward primary care workforce preparation contribute to educational innovation and to national health system strategy.

This paper describes the redesign of COM353, a mandatory four-week Family Medicine and Primary Health Care block delivered to third-year medical students at Alfaisal University. The block was rebuilt around three pedagogical commitments: generalism as a clinical method requiring deliberate teaching, clinical courage as a cultivable capacity for working with uncertainty, and community-based practice as a primary site of clinical learning deserving the same investment as hospital rotations. The account that follows lays out the curricular design and its pedagogical framework, the partnership architecture that made the design implementable (an academic-academic exchange with the University of Toronto Department of Family and Community Medicine and academic-service partnerships with Riyadh PHC Centers), preliminary student-reported outcomes from the first delivered cohort, and lessons for medical schools seeking to function as active partners in PHC transformation rather than as detached producers of graduates.

## 2 Pedagogical framework, principles, and competencies

Three pedagogical commitments define COM353. None is sufficient on its own.

### 2.1 Generalism as a core clinical method

The block treats Family Medicine not as a residual category but as a distinct clinical discipline. Comprehensiveness, continuity, first-contact access, and coordination, the attributes Starfield identified as defining Primary Care, require physicians to integrate biomedical, psychosocial, and preventive reasoning within a single encounter, across the lifespan, and over time (10). What this asks of clinicians is not a simpler form of specialty medicine but a different kind of clinical thinking, and the teaching has to reflect that. The block names generalism as expertise and structures its activities around the cognitive and relational demands generalist practice entails.

### 2.2 Clinical courage as a cultivable capacity

Medical education has often treated uncertainty as a problem to be resolved through more tests, more data, more consultation. In Primary Care, uncertainty is less a problem to solve than a condition to inhabit. COM353 introduces clinical courage as the willingness to engage thoughtfully with patients despite incomplete information, to acknowledge knowledge limits without retreating from responsibility, and to reason carefully rather than defensively. The disposition is cultivated through repeated exposure to ambiguous clinical scenarios, faculty modeling under uncertainty, and the deliberate creation of psychological safety that lets students say “I do not know” without penalty. Reis-Dennis and colleagues’ framework of three corrective virtues (courage, diligence, curiosity) gives the block both conceptual scaffolding and a vocabulary students recognize as different from the language of mastery (6).

### 2.3 Community-based practice as a primary learning site

The third commitment is architectural. COM353 positions Primary Health Care Centers as primary sites of clinical education, deserving the same curricular investment, preparation, and analytical rigor as any tertiary facility. Community visits are framed in advance, structured for active engagement, and debriefed through reflection. The academic department invests in these placements through formal partnerships, ongoing communication with supervising physicians, and explicit alignment of learning objectives with what students actually encounter in community practice.

Two theoretical frameworks anchor the curriculum. Kolb’s experiential learning cycle informs how each PHC placement is sequenced through concrete experience, reflective observation, abstract conceptualization, and active experimentation (11, 12). Dornan and colleagues’ Experience-Based Learning model treats reflection as the mechanism by which clinical experience is converted into capability and identity, with supported participation as the core condition for effective workplace learning (13). Together these frameworks ground the block’s repeated concept-experience-reflection rhythm.

**Figure 1:**
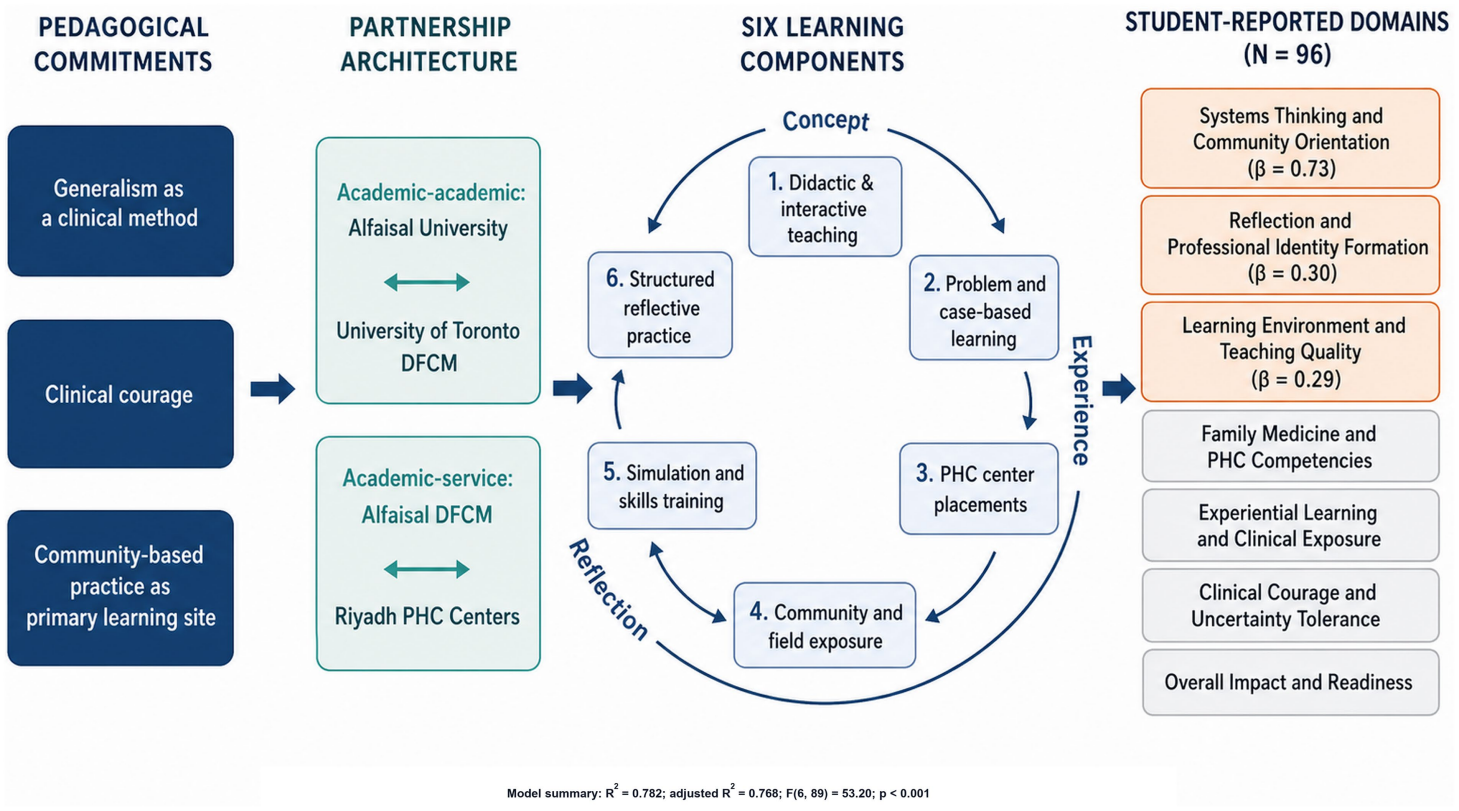
Conceptual architecture of the COM353 Family Medicine and Primary Care block, mapping pedagogical intent to evaluated outcomes. Three pedagogical commitments (generalism as a clinical method, clinical courage, and community-based practice as the primary learning site) anchor the design. Implementation runs through two partnership layers: an academic-academic exchange between Alfaisal University DFCM and the University of Toronto DFCM, and academic-service partnerships between Alfaisal DFCM and Primary Health Care Centers across Riyadh. Six learning components are sequenced as iterative concept-experience-reflection cycles drawing on Kolb’s experiential learning cycle and the Experience-Based Learning framework of Dornan and colleagues. Student-reported outcomes (*N* = 96) span seven *a priori* domains. Three were independent predictors of Overall Impact and Readiness in the multiple linear regression model and are highlighted: Systems Thinking and Community Orientation (*β* = 0.73), Reflection and Professional Identity Formation (*β* = 0.30), and Learning Environment and Teaching Quality (*β* = 0.29). Model fit: R^2^ = 0.782, adjusted R^2^ = 0.768, *F*(6, 89) = 53.20, *p* < 0.001. *β*, standardized regression coefficient; DFCM, Department of Family and Community Medicine; PHC, Primary Health Care.

The competencies the block targets map to these commitments: the four Starfield attributes; recognition of the family physician’s first-contact and coordinating roles; comfort with incomplete information; reflective capacity sufficient to make experience educational; and explicit awareness of healthcare as an interconnected system in which referral pathways, social determinants, and policy decisions shape patient outcomes (Figure 1).

These are, for the most part, primary care competencies in Starfield’s sense, the attributes of the first-contact clinical service rather than the full reach of Primary Health Care as a system. The block teaches them directly and then, through its systems-thinking and community components, sets them inside the wider Primary Health Care architecture of essential public health functions, multisectoral action, and community engagement (2). Building primary care competence at the undergraduate stage is, in this setting, a concrete contribution to Primary Health Care-oriented reform: a system reorganizing around Primary Health Care needs clinicians who are skilled in the first-contact discipline and who grasp how that discipline connects to the structures around it, which is what Saudi Arabia’s Vision 2030 workforce strategy calls for (9).

## 3 Learning environment, objectives, and pedagogical format

### 3.1 Setting and students

Alfaisal University is a private, research-oriented institution in Riyadh. The College of Medicine offers a six-year undergraduate program organized around integrated organ-system blocks, with formal clinical clerkships beginning in year four. COM353 is a mandatory four-week block delivered to all third-year medical students. Its placement is deliberate: by year three, students have a working biomedical foundation but limited sustained exposure to authentic clinical environments and have not yet entered hospital-based clerkships that tend to dominate clinical training and shape professional identity. The block reaches students before identities harden around hospital-centric assumptions.

The students in the cohort were all third-year medical students in the College’s six-year MD program. By the time of the block they had completed the preclinical foundation and the early part of year three, but had not yet begun the hospital-based clerkships that start in year four, and none had undertaken a formal Family Medicine placement before COM353. Within the College, COM353 is institutionally titled “Comprehensive Community Health” and is the third-year block in which Family Medicine and Primary Care are taught. Teaching across the College is delivered in English. The class is mixed and is taught in parallel male and female sections in line with institutional practice, with both sections receiving the same course material, learning objectives, and assessment. Participation in the block is mandatory; participation in the evaluation survey was voluntary and anonymous.

### 3.2 Partnership architecture: faculty, sites, and the Toronto exchange

The block is delivered by faculty of the Department of Family and Community Medicine in collaboration with clinical preceptors at partner Primary Health Care Centers across Riyadh. Two distinct partnership layers underpin its operation, and the deliberate distinction between them is one of the design choices most easily missed in summary descriptions.

#### 3.2.1 Academic-academic partnership

The redesign was catalyzed by sustained scholarly engagement with the Department of Family and Community Medicine at the University of Toronto, including bidirectional faculty visits, joint teaching, and a structured curriculum-development exchange in 2025. The Toronto Department, with more than four decades of postgraduate Family Medicine education and a research culture organized around community-based teaching, contributed an established vocabulary for generalist practice as a discipline, experience operationalizing reflective and experiential pedagogy at scale, and the model of community-based settings as primary learning sites. Alfaisal contributed a Gulf-region undergraduate context, alignment with Vision 2030 workforce priorities, and a working PHC partnership network in Riyadh. The exchange shaped the pedagogical framing of the block, the architecture of community placement, and the language used with both students and preceptors.

#### 3.2.2 Academic-service partnership

COM353 operates through formal partnerships with PHC Centers in Riyadh, not *ad hoc* placement arrangements. The Department maintains site-specific memoranda of understanding, designates clinical leads at each center, and holds preceptor orientation sessions before each block delivery in which the pedagogical framework, expected student behaviors, and reflection prompts are reviewed with supervising physicians. Without that orientation, the gap between what students hear in class and what they observe in clinic undermines coherence. The departmental time required to maintain these relationships is substantial and recurring, and is, in our experience, the single largest unrecognized cost of partnership-based curricula and the most consequential investment in their educational outcomes.

### 3.3 Learning objectives

By the end of the block, students should be able to describe the core attributes of Primary Care and articulate the family physician’s distinct role in the health system, observe and interpret patient-centered, longitudinal, and preventive practice in real primary care encounters, reason productively in clinical uncertainty without resorting to defensive medicine, reflect critically on clinical experiences in ways that link encounters to professional values, and analyze healthcare as an interconnected system with attention to referral pathways, equity, and the social determinants of health.

### 3.4 Pedagogical format

Six learning components are sequenced into iterative concept, experience, reflection cycles across the 4 weeks.

#### 3.4.1 Didactic and interactive teaching

Core sessions introduce the intellectual framework of Family Medicine and Primary Care, covering continuity, comprehensiveness, coordination, first-contact access, preventive practice, patient-centered communication, referral pathways, the social determinants of health, and the physician’s role in community-oriented care. Faculty design these as interactive engagements, posing clinical dilemmas without clean answers and modeling how experienced clinicians reason when information is incomplete. The intent is to establish uncertainty as a normal feature of clinical work before students encounter it in the field.

#### 3.4.2 Primary health care center placements

PHC visits constitute the block’s central experiential component and the most significant departure from the previous curriculum. Students are placed in functioning PHC Centers under family physician supervision, where they follow patient encounters and observe how physicians manage multiple concerns within a single visit, weaving chronic disease management and preventive care into acute presentations. Visits are bracketed by preparatory framing and post-visit guided reflection so that what students observe is interpreted analytically rather than absorbed passively (11, 13).

#### 3.4.3 Community and field exposure

Beyond the PHC Centers, students participate in structured visits to emergency medical services (Saudi Red Crescent), public health facilities, and community health programs, making the systems infrastructure surrounding primary care visible. The component operationalizes the systems thinking commitment, since healthcare cannot be understood as a system through reading alone.

#### 3.4.4 Simulation and skills training

Simulation laboratories provide controlled environments for developing skills relevant to community-based practice, including patient safety scenarios, chronic disease management, communication, and procedural competencies appropriate to primary care. The sessions complement experiential learning by allowing low-stakes practice before similar situations arise in authentic settings.

#### 3.4.5 Problem-based and case-based learning

Structured case discussions bring theoretical knowledge into contact with clinical experience. Cases are drawn from real Primary Care practice and selected to feature ambiguity, competing priorities, and system-level considerations, so that the right answer depends on context, resources, patient preferences, and the physician’s comfort with uncertainty.

#### 3.4.6 Structured reflective practice

Reflection is embedded across the block as a core pedagogical strategy rather than as an assessment artifact. After experiential activities, students complete structured reflective writing that prompts critical examination of encounters that challenged expectations, observations of patient-centered or fragmented care, and emerging professional values (14, 15). Faculty respond with formative feedback that aims to deepen thinking rather than grade compliance. Without structured reflection, community placements risk becoming passive observation; with it, they become developmental experiences that shape how students think about patients, systems, and themselves (13).

### 3.5 Sequencing and integration

Components are sequenced so that conceptual framing precedes experience and reflection follows it, with each pass deepening understanding. Without preparatory framing, a PHC visit becomes tourism; reflection uncoupled from experience drifts into abstraction; didactic teaching unmoored from experiential grounding becomes information that students store and forget.

### 3.6 Curriculum materials and implementation resources

We have assembled the operational materials needed to run the block so that other programs can adopt or adapt it rather than infer it from description alone. The block is reported against the EQUATOR-listed Guideline for Reporting Evidence-based practice Educational interventions and Teaching, which sets out what an account of an educational intervention should contain to be reproducible (16). Four appendices are provided as Supplementary material. Supplementary Appendix S1 reproduces the four-week course schedule as delivered, showing how the thirty teaching sessions, the three problem-based learning cases, and the experiential visits to Primary Health Care Centers and to the Saudi Red Crescent Authority are distributed across the block. Supplementary Appendix S2 sets out the educational philosophy of the block, the formal learning objectives in the knowledge, skills, and professionalism domains, the seven learning modalities, the three PBL case titles, the partner clinical sites, the assessment scheme, and the full lecture list. Supplementary Appendix S3 is the student-facing Experiential Learning Reflection Guidelines, including the required reflection structure, length and formatting requirements, the ethical conduct rules for the visits, and the assessment rubric used for the SRCA and PHC reflections. Supplementary Appendix S4 reproduces the full 42-item survey instrument with its seven-domain mapping and the two closing items. With these materials a reader can reconstruct the curriculum content, the placement structure, the reflective-practice assessment, and the evaluation tool used in this study.

## 4 Preliminary results from the first delivered cohort

### 4.1 Survey instrument

We constructed a 42-item instrument adapted from the Dundee Ready Education Environment Measure (DREEM) and the Groningen Reflection Ability Scale (GRAS), supplemented with items developed for domains central to COM353 but not fully captured by these tools (uncertainty tolerance in PHC settings, experiential learning, and systems thinking). Items were grouped *a priori* into seven domains aligned with the block’s stated objectives: Learning Environment and Teaching Quality (7 items); Family Medicine and PHC Competencies (7); Experiential Learning and Clinical Exposure (6); Clinical Courage and Uncertainty Tolerance (6); Reflection and Professional Identity Formation (6); Systems Thinking and Community Orientation (5); and Overall Impact and Readiness (5). All items used a five-point Likert scale (1 = Strongly Disagree to 5 = Strongly Agree). The survey closed with a global block rating and one open-ended item. The full instrument, with all items grouped by domain, is reproduced in Supplementary Appendix S4.

### 4.2 Administration and ethics

All third-year students completing the block in January 2026 (*n* = 126) were invited to participate. Administration was online, anonymous, and immediately post-block. The study was approved by the Alfaisal University Institutional Review Board and conducted in accordance with the Declaration of Helsinki, with informed consent obtained electronically before the survey opened. Participation was voluntary and no inducement was offered.

### 4.3 Analysis

Descriptive statistics were calculated at item and domain level. Internal consistency was assessed by Cronbach’s alpha. Effect sizes were calculated as Cohen’s d against the scale midpoint (3.0), interpreted using both Cohen’s conventional thresholds and the education-specific benchmarks proposed by Kraft. Multiple linear regression with the six predictor domains entered simultaneously tested which domains independently predicted Overall Impact and Readiness. Regression assumptions (linearity, normality of residuals, homoscedasticity, and multicollinearity by variance inflation factors) were checked before interpretation. Analyses were performed in IBM SPSS Statistics version 29 (IBM Corp., Armonk, NY).

### 4.4 Findings

Of 126 enrolled students, 96 completed the survey, a response rate of 76.2% that is consistent with the range reported in recent medical education survey research (17, 18); we acknowledge that non-response bias cannot be excluded. All responses were complete. Students reported favorable perceptions across all seven domains, with mean scores from 3.90 to 4.27 on the five-point scale and effect sizes (Cohen’s d) of 0.92 to 1.58, exceeding conventional thresholds for large effects (19). By education-specific benchmarks, where d = 0.6 is considered large, the magnitudes are exceptional (20). Agreement rates ranged from 63.7 to 80.7%. Internal consistency was excellent at the domain level (Cronbach’s alpha 0.940 to 0.974) and overall (alpha = 0.988); we discuss the implications of the latter in the constraints section.

To address the source of the exceptionally high overall internal consistency directly, we computed corrected item-total correlations (CITC) for all 42 items. CITC values ranged from 0.675 to 0.900 (mean 0.809, median 0.809). Forty-one of the forty-two items (98%) had a CITC of 0.70 or higher, and twenty-three items (55%) had a CITC of 0.80 or higher; no item fell below 0.50. The mean inter-item correlation across all 861 unique item pairs was 0.664, well above the upper bound of approximately 0.50 conventionally recommended for items intended to measure distinct facets of a unidimensional scale (21). The uniformity and the magnitude of these correlations indicate that the items, despite mapping to seven *a priori* domains, are largely indexing one broad construct, most plausibly a global positive evaluation of the block. We therefore treat the domain-level regression coefficients reported in Table 1 as exploratory descriptions of patterns within a single broad construct rather than as evidence of seven empirically independent factors, and we return to this in Section 6. Per-item CITC values are provided in Supplementary Appendix S4.

Looking at the domains in turn, Family Medicine and PHC Competencies stood out, with the highest mean (4.27) and an agreement rate of 80.7%. Continuity of care drew 84.4% agreement and the first-contact role 81.2%, suggesting the block communicated the core intellectual content of generalist practice effectively. Clinical Courage and Uncertainty Tolerance scored slightly lower (mean 4.01, agreement 67.5%), but the underlying picture was more interesting than the headline figures: roughly two thirds of students reported greater comfort with ambiguity, and the item on thoughtful reasoning rather than defensive medicine drew 76.0% agreement, which is not a trivial shift for a pre-clerkship cohort. The regression analysis was perhaps the most informative part of the data set. Systems Thinking and Community Orientation emerged as the strongest predictor of overall block satisfaction (*β* = 0.727, *p* < 0.001), with Reflection and Professional Identity Formation (*β* = 0.295, *p* = 0.002) and Learning Environment (*β* = 0.293, *p* = 0.013) also independently predictive. The six predictors accounted for 78.2% of the variance, and variance inflation factors stayed below 5. The regression model and domain-level statistics are presented in Tables 1, 2, respectively.

**Table 2:** Domain-level descriptive statistics, internal consistency, and effect sizes (*N* = 96).

Domain	Items	Mean (SD)	Agree %	Cronbach α	Cohen’s d	95% CI (mean)
Learning Environment and Teaching Quality	7	4.05 (0.86)	69.6	0.952	1.22	[3.88, 4.22]
Family Medicine and PHC Competencies	7	4.27 (0.80)	80.7	0.954	1.58	[4.11, 4.43]
Experiential Learning and Clinical Exposure	6	4.04 (0.91)	70.0	0.962	1.15	[3.86, 4.22]
Clinical Courage and Uncertainty Tolerance	6	4.01 (0.85)	67.5	0.940	1.19	[3.84, 4.18]
Reflection and Professional Identity Formation	6	3.90 (0.98)	63.7	0.974	0.92	[3.70, 4.10]
Systems Thinking and Community Orientation	5	4.10 (0.85)	72.5	0.951	1.29	[3.93, 4.27]
Overall Impact and Readiness	5	4.13 (0.89)	75.4	0.962	1.27	[3.95, 4.31]

All items used a five-point Likert scale (1 = Strongly Disagree, 5 = Strongly Agree). Agree % combines Agree and Strongly Agree responses. Cohen’s d calculated against the scale midpoint (3.0); all d values exceed Cohen’s threshold of 0.8 for large effects. 95% CI = 95% confidence interval for the domain mean. Overall instrument Cronbach’s α = 0.988.

**Table 1:** Multiple linear regression: predictors of overall block satisfaction (*N* = 96).

Predictor	B	SE B	*β* (standardized)	*t*	*p*
(Constant)	0.122	0.182	n/a	0.67	0.503
Systems Thinking and Community Orientation	0.689	0.082	0.727	8.40	< 0.001
Reflection and Professional Identity Formation	0.243	0.077	0.295	3.16	0.002
Learning Environment and Teaching Quality	0.275	0.108	0.293	2.55	0.013
Family Medicine and PHC Competencies	−0.123	0.115	−0.122	−1.07	0.288
Experiential Learning and Clinical Exposure	−0.057	0.083	−0.066	−0.69	0.493
Clinical Courage and Uncertainty Tolerance	0.040	0.090	0.044	0.45	0.654

Dependent variable: Overall Impact and Readiness domain mean. Six predictor domains entered simultaneously. R^2^ = 0.782; adjusted R^2^ = 0.768; *F*(6, 89) = 53.20, *p* < 0.001. Variance inflation factors all < 5; no multicollinearity concerns.

## 5 Discussion

The COM353 redesign is an attempt to take seriously what the medical education literature has been saying for some time, that Primary Health Care needs dedicated, experiential, reflective curricular attention if students are to understand it as a discipline rather than absorb it as an afterthought. The student-reported outcomes are consistent with the design’s intent. The contribution of this paper, however, lies less in the survey numbers than in the design principles they reflect.

### 5.1 Systems thinking as the integrating competency

The regression result, that systems thinking outpaced content knowledge, experiential exposure, and uncertainty tolerance as a predictor of overall satisfaction, takes some unpacking. The simplest reading would be that students value big-picture thinking, but the Health Systems Science literature points to something more specific. Systems thinking functions as a linking domain, connecting clinical competencies to one another and to the broader healthcare context (7, 8). Students who develop systems awareness acquire a framework that lets the other competencies cohere rather than simply adding one more to the list. Gonzalo and colleagues’ subsequent work on systems citizenship, describing physicians as participants in health system evolution, gives identity-level language to that integration (22). For design, systems thinking is best treated as the connective tissue holding everything else together, not as one domain among many.

This finding has practical weight in the Saudi context. Vision 2030 demands physicians who can work inside a health system in active redesign, and graduates who think in systems, who understand how primary care interfaces with secondary and tertiary services, how community health connects to national policy, and how workforce decisions shape population outcomes, are what this transformation requires (9). That a pre-clerkship block can begin cultivating the orientation is encouraging.

### 5.2 The paradox of reflection

Reflection showed the lowest absolute scores yet emerged as the second strongest predictor of overall evaluation, a pattern well documented in medical education but rarely discussed in curriculum design papers (23, 24). The likeliest explanation is vertical. Reflective capacity matures from descriptive surface engagement toward more critical examination of assumptions, values, and identity (14), and not every student reaches the deeper levels in a single four-week block. Students who do engage meaningfully appear to derive disproportionate benefit, however, and the practical implication is that quality of reflection matters more than quantity. The design priority becomes the conditions under which meaningful reflection becomes possible, including psychological safety, formative feedback, and explicit linking of prompts to specific clinical experiences. Cruess and colleagues’ framework on professional identity formation, which positions reflection as central to the active internalization of professional values, adds further weight (25, 26).

### 5.3 Clinical courage as a curricular priority

That two-thirds of students reported greater comfort with uncertainty after a pre-clerkship block is, in itself, worth noting. For most of them, this was a first structured engagement with ambiguity as a feature rather than a bug of clinical practice. The result lines up with Patel and colleagues’ scoping review, which found that 22 of 24 educational interventions targeting uncertainty tolerance reported positive impact (5), and it sits comfortably alongside Hillen and colleagues’ integrative model, which identifies ambiguity, probability, and complexity as distinct sources of uncertainty (4). COM353 engages all three: ambiguity through undifferentiated PHC presentations, probability through diagnostic reasoning under incomplete information, and complexity through systems-level engagement. The clinical courage framing gave the block a vocabulary students recognized as different from the dominant educational language of mastery (6), and giving the capacity to act despite not knowing a name appears to give students permission to be uncertain and a way of talking about it.

### 5.4 Experiential learning and community placement

The large effect size for the experiential learning domain (d = 1.15) supports positioning PHC visits as the block’s central component. Beyond skills development, community-based learning makes visible a model of clinical practice many students would otherwise never encounter. Hospital-based clerkships, however well delivered, present an acute, episodic, specialist-driven version of medicine, while PHC placements offer a longitudinal, comprehensive, generalist alternative embedded in community. Students who experience both are better positioned to make informed decisions about where they fit. When PHC visits are framed as optional enrichment, the implicit message is that community-based practice is similarly optional; framing them as central, with the same standards of intellectual rigor as anything else, changes the message (27).

### 5.5 The partnership architecture as curriculum

The two partnership layers in Section 3.2 are part of the curriculum rather than external supports for it. The Toronto exchange supplied conceptual vocabulary, pedagogical models tested in another setting, and a posture of generalism as legitimate scholarly territory. The PHC center partnerships supplied the clinical reality any curriculum aspiring to take Primary Care seriously requires, including patients followed over time, continuity of care, and the system pressures clinicians actually contend with. The Lancet Commission on health professionals for a new century identified inter-institutional collaboration as a structural condition for the curricular redesign global health systems now require, and the COM353 experience supports that claim at block level (28).

The pre-clerkship orientation described here joins a small but growing body of regional accounts on undergraduate Family Medicine and Primary Health Care education. Sulaiman, Shorbagi, and Guraya (29) used Kern’s six-step model to develop and longitudinally evaluate the Family Medicine program at the College of Medicine, University of Sharjah, drawing on eight academic years and 804 medical students; their account converges with ours on the recurring challenges of dedicated faculty time, adjunct preceptor capacity at PHC sites, and the need for sustained curricular visibility for Family Medicine within the wider undergraduate program. Daher-Nashif et al. (30) reported a qualitative account of student-led Family Medicine clinics at Qatar University’s College of Medicine, delivered through a partnership with the Primary Health Care Corporation; students described the eight-week rotation as a transitional and transformative stage in their developing professional identity, a pattern compatible with what we observed for the Reflection and Professional Identity Formation domain. Earlier work at King Saud bin Abdulaziz University for Health Sciences in Riyadh examined the attitudes of 308 medical students toward Family Medicine as a future career specialty, identifying both interest and structural barriers that pre-clerkship exposure could plausibly modify (31). Across these accounts and our own, the recurring elements are explicit engagement with PHC as a primary clinical site, sustained academic-clinical partnerships, and the difficulty of sustaining preceptor capacity. The convergence suggests that the curricular elements we report are not idiosyncratic and that a regional dialog on undergraduate PHC education is now beginning to coalesce.

### 5.6 Practical implications

A few implications stand out. The most basic is also easy to underestimate: an explicit pedagogical framework changes how students experience a block, and without one, redesign tends to produce better-organized teaching of the same old content. Closely related is the question of partnerships with delivery sites, which are pedagogical necessities rather than logistical conveniences, with faculty time on preceptor orientation and ongoing communication needing to be planned for at the front end. Academic-academic exchanges follow similar logic, delivering returns disproportionate to their cost when oriented around a concrete curricular product, less so when they remain at the level of a memorandum of intent. The data themselves push toward one further point: systems thinking deserves curriculum design weight that may exceed its formal teaching share. If it is functioning as connective tissue, treating it as one box among seven on a syllabus will under-deliver its value.

## 6 Acknowledgment of constraints

The design and evaluation reported here have constraints worth naming honestly.

### 6.1 Conceptual constraints

The pedagogical commitments are not novel in isolation. Generalism, experiential learning, and systems thinking each have established literatures, and we are not the first to bring them together in a single block. Our claimed contribution is in the integration and in the specificity of its application to a Gulf-region undergraduate program.

### 6.2 Methodological constraints

The cross-sectional, single-cohort design captures perceptions at one time point and cannot test whether reported gains persist into clerkship and practice. Self-report data carry standard risks of social desirability, even with anonymous administration. The absence of a comparison group limits causal inference. A formal, free-standing needs assessment with primary data from students, faculty, and community stakeholders was not carried out before the redesign. The design drew instead on the documented under-representation of generalist and community-based practice in undergraduate training (3), on the structured curriculum-development exchange with the University of Toronto Department of Family and Community Medicine, and on the national workforce priorities set out under Saudi Arabia’s Vision 2030 (9). The post-block survey reported here now provides a first cohort-level baseline against which later cohorts and design changes can be compared. The instrument represents adaptation of existing tools rather than independent validation, and the exceptionally high overall internal consistency (*α* = 0.988) raises a legitimate possibility of item redundancy. Tavakol and Dennick noted that alpha values exceeding 0.90 may indicate overlapping constructs (32); future iterations of the survey will examine whether the instrument can be streamlined without losing domain-level specificity.

Consistent with the corrected item-total correlation pattern reported in Section 4.4, where most items showed CITC values in the 0.70 to 0.90 range and the mean inter-item correlation was 0.664, the seven *a priori* domains overlap substantially in empirical performance. The domain-level regression coefficients in Table 1 should therefore be read as exploratory and as descriptive of patterns within a single broad construct, not as evidence of independent domain-specific effects. This does not undermine the substantive observation that systems-thinking content covaried most strongly with the overall evaluation, but it does mean that statements about relative domain contributions need appropriate caution until the instrument is refined and re-evaluated on a cohort large enough to support confirmatory factor analysis.

### 6.3 Resource and sustainability constraints

Partnership-based curricula are not low-cost. The model requires ongoing departmental investment in preceptor orientation, site visits, joint teaching with international partners, and administrative scaffolding around memoranda of understanding and student logistics. Schools considering replication should budget for these recurring costs rather than treating them as one-off setup expenses. Faculty turnover, leadership change at partner sites, and shifting institutional priorities all threaten continuity, and the academic-academic component depends on personal relationships that take years to build and only months to lose. Institutional rather than individual ownership of the partnership has been our way of mitigating this fragility.

### 6.4 Future directions

Several lines of work follow directly from this report. The most immediate is longitudinal follow-up of the cohort into clerkships and beyond, which would test whether the perceptual gains reported here in generalist competence, systems thinking, and clinical courage translate into observable behavior. Alongside that, exploratory and confirmatory factor analysis on accumulated cohorts would let us refine the instrument and address the redundancy concern flagged by the high overall alpha. Multi-site replication, ideally with comparator institutions still running the previous block design, would let the field move from descriptive to comparative evidence about partnership-based curricular redesign, which is where this literature ultimately needs to go.

What the evaluation establishes is that a curriculum designed with explicit educational philosophy, supported by academic-service partnerships, and combining experiential immersion with reflective practice and engagement with clinical uncertainty was associated with a coherent, favorable response across the competencies it was built to develop. The alignment between pedagogical intent and student perception provides a foundation on which longer-term and multi-site evidence can now be sought.

## 7 Conclusion

This paper has described the redesign of a pre-clerkship Family Medicine and Primary Care block grounded in three pedagogical commitments: generalism as a clinical method, clinical courage as a cultivable disposition for working with uncertainty, and community-based practice as a primary site of clinical learning. Preliminary student-reported outcomes from 96 third-year students suggest the design is functioning as intended, with systems thinking emerging as the dominant predictor of perceived value. The partnerships that make the model possible (academic department to PHC sites, Alfaisal to Toronto, educational objectives to Vision 2030 priorities) are constitutive of the educational intervention rather than incidental to it.

If we have to distill the experience to its transferable elements, a few stand out. Pedagogical commitments should be articulated before content is redesigned, because commitments shape architecture in ways content alone does not. Community-based settings deserve the status of primary clinical learning sites rather than supplementary placements, since structural messages travel further than spoken ones. Where academic-academic exchange is part of the design, it works best as a tool oriented around a concrete curricular product. The recurring cost of partnership maintenance has to be planned for from the outset rather than absorbed by goodwill, which tends to run out. And systems thinking is best understood not as a content area but as a connective domain whose educational return comes through linkage to the others. Curriculum design is, in this sense, itself a form of academic-service partnership, and one of the practical ways medical schools can contribute to the PHC transformation their countries require.

## Data Availability

The original contributions presented in the study are included in the article/Supplementary material, further inquiries can be directed to the corresponding authors.

## Supplementary material

SUPPLEMENTARY DATA SHEET 1 Supplementary Appendices S1 to S4. S1, the four-week COM353 course schedule as delivered; S2, the educational philosophy, learning objectives, learning modalities, problem-based learning case titles, partner clinical sites, assessment scheme, and full lecture list; S3, the student-facing Experiential Learning Reflection Guidelines with the assessment rubric; S4, the 42-item post-block survey instrument with its seven-domain mapping, followed by the per-item corrected item-total correlations (S4.1).
