# Supplementary material for: Designing for generalism, courage, and systems: a curriculum scholarship account of an integrated family medicine and primary care block
Source: Front Med (Lausanne). 2026 Aug 5;13:1870368. doi: 10.3389/fmed.2026.1870368 (PMC13486183; doi:10.3389/fmed.2026.1870368)
Supplement: Supplementary file 1 [file Data_Sheet_1.DOCX]

**Supplementary Material**

*Designing for Generalism, Courage, and Systems: A Curriculum Scholarship Account of an Integrated Family Medicine and Primary Care Block*

Frontiers in Medicine, Healthcare Professions Education | Manuscript ID 1870368

Alghalyini, Zaidi, Khaled, Alswes, and Batty

This file provides the operational materials for the COM353 block so that the design can be implemented or adapted by other programs. Materials are reproduced from the formal course documentation maintained by the Department of Family and Community Medicine at Alfaisal University College of Medicine, in keeping with the Guideline for Reporting Evidence-based practice Educational interventions and Teaching (GREET; Phillips et al., 2016).

Contents: Appendix S1, the four-week course schedule as delivered; Appendix S2, the educational philosophy, formal learning objectives, learning modalities, problem-based learning case titles, partner clinical sites, assessment scheme, and full lecture list; Appendix S3, the student-facing Experiential Learning Reflection Guidelines used for the SRCA and PHC visit reflections; Appendix S4, the 42-item post-block evaluation instrument, followed by the per-item corrected item-total correlations (S4.1).

A note on naming: within the College, COM353 is institutionally titled "Comprehensive Community Health" and is the third-year block in which Family Medicine and Primary Care are taught. The block is referred to throughout the manuscript by its disciplinary scope, while the supplementary materials use the formal institutional title.

**Appendix S1. COM353 Course Schedule (Four Weeks)**

The schedule below reproduces the four-week course timetable as documented in the formal course syllabus and as delivered to the cohort that completed the block in early January 2026. The College runs parallel male and female sections that share course material, learning objectives, and assessment; the schedule shows the activity for each time slot, with the same content delivered to both sections. Three introductory sessions (Lect 1, 2, and 3, covering social determinants of health, the healthcare process, and the Health Promoting Practices Exhibition visit) were delivered in an orientation week immediately preceding Week 1. The full lecture list is provided in Appendix S2.

Block dates: 7 December 2025 to 1 January 2026, with the final examination week immediately following. Teaching venues: Male section, Room M1.013; Female section, Room MS1.024. Working days: Sunday through Thursday.

**Week 1 (7 to 11 December 2025)**

| **Time** | **Sunday (7 Dec)** | **Monday (8 Dec)** | **Tuesday (9 Dec)** | **Wednesday (10 Dec)** | **Thursday (11 Dec)** |
| --- | --- | --- | --- | --- | --- |
| **8:00 to 8:50** | SDL / Lect 4: Value-Based and Team-Based Care | Lect 9: Choosing Wisely and Deprescribing / Lect 7: Public Health Regulations and Legislation vs Policy | - | SDL / Lect 10: Vaccination Across the Lifespan | Cross-block: Evidence Based Medicine (EBM 354) |
| **9:00 to 9:50** | Lect 4: Value-Based and Team-Based Care / Lect 5: Primary, Secondary, and Tertiary Care | Lect 7: Public Health Regulations and Legislation vs Policy / Lect 9: Choosing Wisely and Deprescribing | Experiential Learning: KFMC PHC Visit (Lect 25) / SRCA Visit (Lect 26) | SDL | - |
| **10:00 to 10:50** | Lect 5: Primary, Secondary, and Tertiary Care / Lect 6: Sustainability in Healthcare | PBL 1 (Chronic Disease Management in the Community), Session 1 | Experiential Learning continues | Lect 10: Vaccination Across the Lifespan / SDL | Cross-block: EBM 354 and PRO 355 |
| **11:00 to 11:50** | Lect 6: Sustainability in Healthcare / SDL | PBL 1, Session 1 (continued) | Experiential Learning continues | - | - |
| **12:00 to 1:00** | Prayer and Lunch | Prayer and Lunch | Prayer and Lunch | Prayer and Lunch | Prayer and Lunch |
| **1:00 to 1:50** | SDL / Cross-block: PRO 355 | SDL / Cross-block: MIF 356 | Experiential Learning: SRCA / KFMC PHC (continued) | Lect 11: Disease Prevention and Health Promotion Screening | PBL 1, Session 2 |
| **2:00 to 2:50** | SDL | SDL | - | Lect 12: Communicable Disease Control: Lessons from COVID-19 | - |
| **3:00 to 3:50** | Group Review Class (GRC) | Cross-block: MIF 356 / SDL | SDL | Lect 11 / Lect 12 (parallel section) | Lect 13: Non-Communicable Diseases and WHO STEPS Tools / SDL |
| **4:00 to 5:00** | - | - | SDL | Lect 12 (parallel section) | Lect 13 (parallel section) / SDL |
| **5:00 to 6:00** | - | GRC | GRC | GRC | - |

**Week 2 (14 to 18 December 2025)**

| **Time** | **Sunday (14 Dec)** | **Monday (15 Dec)** | **Tuesday (16 Dec)** | **Wednesday (17 Dec)** | **Thursday (18 Dec)** |
| --- | --- | --- | --- | --- | --- |
| **8:00 to 8:50** | Cross-block: MIF 356 | SDL | Experiential Learning: KFMC PHC (Lect 27) / SRCA (Lect 28) | SDL | Cross-block: EBM 354 and PRO 355 |
| **9:00 to 9:50** | - | SDL | Experiential Learning continues | Lect 17: Guest Speaker | - |
| **10:00 to 10:50** | - | PBL 2 (Patient Safety and Health Promotion in Primary Care), Session 1 | Experiential Learning continues | Lect 18: Patient Safety in Family and Community Medicine | Cross-block: EBM 354 and PRO 355 |
| **11:00 to 11:50** | - | PBL 2, Session 1 (continued) | Experiential Learning continues | Lect 18 (parallel section) | - |
| **12:00 to 1:00** | Prayer and Lunch | Prayer and Lunch | Prayer and Lunch | Prayer and Lunch | Prayer and Lunch |
| **1:00 to 1:50** | Cross-block: PRO 355 / Community Engagement in Action | Lect 15: Cancer Screening and Prevention Strategies / MIF 356 | SDL / Experiential Learning continues | PRO 355 / Lect 18 (parallel) | PBL 2, Session 2 |
| **2:00 to 2:50** | Community Engagement in Action / PRO 355 | - | Lect 16: Environmental Health / Lect 29: Clinical Courage, Identity, and Uncertainty | - | - |
| **3:00 to 3:50** | GRC | Cross-block: MIF 356 / Lect 15: Cancer Screening (parallel section) | Lect 29 / Lect 16 (parallel section) | Lect 19 / SDL | SDL |
| **4:00 to 5:00** | - | - | SDL | SDL / Lect 19 (parallel) | SDL |
| **5:00 to 6:00** | - | GRC | GRC | GRC | - |

**Week 3 (21 to 25 December 2025)**

| **Time** | **Sunday (21 Dec)** | **Monday (22 Dec)** | **Tuesday (23 Dec)** | **Wednesday (24 Dec)** | **Thursday (25 Dec)** |
| --- | --- | --- | --- | --- | --- |
| **8:00 to 8:50** | Cross-block: EBM 354 | Lect 20: Digital Health and AI in Primary Care / Lect 21: Implicit Bias | SDL | Block Review Session (Dr. Alghalyini, Dr. Zaidi, Dr. Khaled) | Cross-block: EBM 354 and PRO 355 |
| **9:00 to 9:50** | - | Lect 21 / Lect 20 (parallel section) | Lect 24: Media Advocacy and Infodemic | Block Review (continued) | - |
| **10:00 to 10:50** | - | PBL 3 (Digital Health and Innovation in Primary Care), Session 1 | Lect 23: Guest Speaker | Block Review (continued) | Cross-block: EBM 354 and PRO 355 |
| **11:00 to 11:50** | - | PBL 3, Session 1 (continued) | Lect 23 (continued) | Block Review (continued) | - |
| **12:00 to 1:00** | Prayer and Lunch | Prayer and Lunch | Prayer and Lunch | Prayer and Lunch | Prayer and Lunch |
| **1:00 to 1:50** | Cross-block: PRO 355 / Lect 8: Global Health from Low- to High-Income Country Perspectives | SDL | Lect 30: Space Medicine | PRO 355 / SDL | PBL 3, Session 2 |
| **2:00 to 2:50** | Lect 8 / PRO 355 | Lect 22: Reproductive Care / SDL | Lect 8 (Guest) | - | - |
| **3:00 to 3:50** | GRC | SDL / Lect 22 (parallel section) | SDL | SDL | SDL |
| **4:00 to 5:00** | - | SDL | SDL | SDL | SDL |
| **5:00 to 6:00** | - | GRC | GRC | GRC | - |

**Week 4 (28 December 2025 to 1 January 2026): Consolidation and Examination**

The final week of the block was dedicated to self-directed study and the final examination, with the COM353 final examination held on the date set by the College examination schedule. Cross-block sessions for parallel courses (EBM 354, PRO 355, MIF 356) continued during this week as scheduled by the College.

Abbreviations used in the schedule. SDL: Self-Directed Learning. PBL: Problem-Based Learning. GRC: Group Review Class. KFMC: King Fahad Medical City. SRCA: Saudi Red Crescent Authority. EBM 354, PRO 355, MIF 356: parallel third-year blocks delivered alongside COM353. Where two activities are separated by a slash, the first applies to the male section and the second to the female section, with both sections receiving equivalent content over the week.

**Appendix S2. Educational Philosophy, Learning Objectives, Modalities, and Assessment**

This appendix reproduces the formal curriculum-document content for the COM353 block: the educational philosophy of the block, the documented learning objectives across the knowledge, skills, and professionalism domains, the learning modalities through which the block is delivered, the titles of the three problem-based learning cases, the partner clinical sites, the assessment and evaluation scheme, and the full list of teaching sessions.

**S2.1 Educational philosophy of the block**

COM353 is designed around the principles of Family Medicine as a discipline of clinical generalism, population accountability, and patient-centered care. The block introduces students to the continuum between individual clinical encounters and population health, emphasizing prevention, safety, equity, and sustainability. The educational approach reflects internationally accepted principles of undergraduate Family Medicine education set out by the College of Family Physicians of Canada (CFPC), the World Organization of Family Doctors (WONCA), and the Royal College of General Practitioners (RCGP), while remaining grounded in Saudi healthcare priorities, national policies, and the Vision 2030 transformation agenda. The block promotes early professional identity formation by positioning family physicians as clinicians, advocates, collaborators, and system stewards.

The educational pillars of the block are: integration of clinical medicine and public health; early exposure to primary care systems; application of patient safety and quality principles; emphasis on health promotion and prevention; and development of reflective practice and professionalism.

**S2.2 Learning objectives**

***Knowledge and understanding***

- K.01 Describe the major social, behavioral, and environmental determinants influencing population health.
- K.02 Describe the structure and processes of the Saudi primary care system and its role in public health.
- K.03 Describe common public health problems and chronic diseases in Saudi Arabia and their prevention strategies.
- K.04 Explain principles of health promotion, disease prevention, and screening across the lifespan.
- K.05 Describe global and national frameworks (for example, WHO and the SDGs) and their relevance to the Saudi context.
- K.06 Explain key public health laws, policies, and ethical considerations guiding family and community medicine practice.
- K.07 Describe concepts of sustainability, cost-effectiveness, and value-based care within health systems.
- K.08 Explain how evidence-based practice and implementation science support primary care transformation.
- K.09 Discuss the role of digital health and AI innovations in modernizing primary care in Saudi Arabia.

***Skills***

- S.01 Diagnose and manage common community-based diseases using integrated clinical and public health approaches.
- S.02 Apply patient safety principles to reduce medical errors in outpatient and community settings.
- S.03 Apply occupational and environmental health concepts in evaluating communicable and non-communicable diseases.

***Values and professionalism***

- V.01 Appreciate the pivotal role of family physicians as health advocates and community resources.
- V.02 Demonstrate integrity, accountability, and professionalism in academic and clinical activities.
- V.03 Reflect on empathy, cultural competence, and the importance of comprehensive patient-centered care.

**S2.3 Learning modalities**

The block spans four intensive weeks and employs a blended, competency-based learning model. Each modality is aligned with course learning outcomes and assessment methods. The modalities used are:

- Interactive lectures with integrated case discussions.
- Problem-based learning (PBL) tutorials, organized in three cases delivered across the block.
- Self-directed learning (SDL).
- Experiential field visits to Second Health Cluster Primary Health Care Centers and to the Saudi Red Crescent Authority (SRCA).
- Office-based procedures and "how-to" skills sessions.
- Reflection exercises and faculty-facilitated debriefs.
- Guest expert sessions.

**S2.4 Problem-based learning case titles**

- PBL 1: Chronic Disease Management in the Community.
- PBL 2: Patient Safety and Health Promotion in Primary Care.
- PBL 3: Digital Health and Innovation in Primary Care.

**S2.5 Partner clinical sites for experiential learning**

Experiential learning is delivered through formal partnerships with two complementary partner organizations, giving students exposure to primary care delivery on one side and to the prehospital and emergency-response system on the other.

- Second Health Cluster Primary Health Care Centers, Riyadh: longitudinal, comprehensive, and continuity-oriented primary care delivery (visits Lect 25 and Lect 27).
- Saudi Red Crescent Authority (SRCA): prehospital emergency response, systems coordination, and community engagement (visits Lect 26 and Lect 28).

**S2.6 Assessment and evaluation scheme**

| **Component** | **Weight** | **Domain** |
| --- | --- | --- |
| Experiential learning reflections (SRCA and PHC visit submissions) | 5% | Values and Professionalism (V.01 to V.03) |
| Problem-based learning sessions (three cases) | 5% | Knowledge, Skills, Professionalism |
| Final written examination | 90% | Knowledge and Skills |
| Total | 100% |  |

**S2.7 Full lecture list**

| **Session** | **Title** |
| --- | --- |
| **Lect 1** | Introduction to COM353 and Social Determinants of Health (SDOH) |
| **Lect 2** | Health Care Process and Patient Experience |
| **Lect 3** | Activities: Students Visit to the Health Promoting Practices (HPP) Exhibition |
| **Lect 4** | Value-Based Care and Team-Based Care |
| **Lect 5** | Primary, Secondary, and Tertiary Care |
| **Lect 6** | Sustainability in Healthcare: Integrating environmentally sustainable practices into public health and clinical care |
| **Lect 7** | Public Health Regulations and Legislation vs Policy |
| **Lect 8** | Global Health from Low- to High-Income Country Perspectives |
| **Lect 9** | Choosing Wisely and Deprescribing |
| **Lect 10** | Vaccination Across the Lifespan |
| **Lect 11** | Principles of Disease Prevention and Health Promotion Screening |
| **Lect 12** | Communicable Disease Control: Lessons from COVID-19 |
| **Lect 13** | Non-Communicable Diseases and WHO STEPS Tools |
| **Lect 14** | Guest Speaker |
| **Lect 15** | Cancer Screening and Prevention Strategies |
| **Lect 16** | Environmental Health |
| **Lect 17** | Guest Speaker |
| **Lect 18** | Patient Safety: Principles and Applications in Family and Community Medicine |
| **Lect 19** | Developing Clinical Courage, Building Personal Identity, and Working with Uncertainty: Family Medicine as the Specialists of Generalism |
| **Lect 20** | Digital Health and AI in Primary Care: Implications for Saudi Arabia |
| **Lect 21** | Implicit Bias |
| **Lect 22** | Reproductive Care |
| **Lect 23** | Guest Speaker |
| **Lect 24** | Media Advocacy and Infodemic: Physician Influence on Policy |
| **Lect 25** | Experiential Learning: KFMC Primary Health Care Visit |
| **Lect 26** | Experiential Learning: Saudi Red Crescent Authority (SRCA) Visit |
| **Lect 27** | Experiential Learning: KFMC Primary Health Care Visit |
| **Lect 28** | Experiential Learning: Saudi Red Crescent Authority (SRCA) Visit |
| **Lect 29** | Developing Clinical Courage, Building Personal Identity, and Working with Uncertainty (parallel-section delivery) |
| **Lect 30** | Space Medicine |

**Appendix S3. Experiential Learning Reflection Guidelines (Student-Facing Document)**

This appendix reproduces the Experiential Learning Reflection Guidelines distributed to students for the COM353 block. The reflections are submitted by every student for both experiential visits, count toward the continuous-assessment component of the block, and are assessed within the Values and Professionalism domain (V.01 to V.03). The guidelines are presented here as students receive them.

**S3.1 Overview**

As part of COM353, students participate in experiential learning visits to (1) the Saudi Red Crescent Authority (SRCA) and (2) Primary Health Care Centers (PHCs) under the Second Health Cluster. Each visit requires a separate structured reflection submission. These reflections contribute to the continuous assessment (5%) and assess learning in the Values and Professionalism domain (V.01 to V.03).

**S3.2 Learning objectives of the reflection**

By completing the reflections, students should demonstrate:

- An understanding of health systems and service delivery.
- Insight into interprofessional teamwork and patient flow.
- Appreciation of patient safety, communication, and professionalism.
- An ability to connect theoretical concepts to real-world practice.
- Reflective capacity and professional insight.

**S3.3 Required reflections**

Students must submit two separate reflections:

A. SRCA Visit Reflection. Focus: prehospital care, emergency response, systems coordination, and community engagement.

B. PHC Visit Reflection. Focus: primary care delivery, continuity of care, patient experience, and preventive services.

Each reflection is assessed independently.

**S3.4 Format and submission requirements (mandatory)**

***File format***

- One Word or PDF file per visit.
- File-name format: COM353_[SRCA or PHC]_Reflection_StudentID.docx

***Length***

- 500 to 700 words per reflection, excluding cover page and images.

***Font and layout***

- Font: Times New Roman or Arial, size 11 or 12.
- Line spacing: 1.15 or 1.5.
- Margins: normal (2.5 cm).

**S3.5 Required structure (must follow exactly)**

***1. Cover Information (not counted in word limit)***

- Student Name.
- Student ID.
- Section (Male or Female).
- Visit Type (SRCA or PHC).
- Date of Visit.

***2. Section A: Description of the Visit (approximately 150 words)***

Briefly describe the setting and organization, services observed, roles of healthcare professionals, and patient flow and workflow. Objective description, with no analysis yet.

***3. Section B: Key Learning Points (approximately 200 to 250 words)***

Discuss what was learned about the healthcare system, how care is coordinated, observed patient safety practices, and the differences between theory and practice.

***4. Section C: Reflection and Professional Insight (approximately 150 to 200 words)***

Reflect on how this visit influenced your understanding of family and community medicine, the professional behaviors observed, the skills or attitudes you aim to develop, and the relevance of the visit to your future clinical training.

***5. Section D: Visual Evidence (mandatory for PHC, optional for SRCA)***

- PHC Visit: upload one to two photographs of the facility or experience. No patients, charts, staff faces, or identifiers are allowed.
- SRCA Visit: photographs are optional, only if permitted on site.
- Each image must include a brief caption (one to two lines).

**S3.6 Ethical and professional rules**

- No patient identifiers.
- No photos of patients or medical records.
- No photos of staff faces without permission.
- No confidential or operationally sensitive information.

**S3.7 Assessment criteria (5% of block grade)**

| **Criterion** | **Weight** |
| --- | --- |
| Completion and adherence to structure | 20% |
| Depth of reflection and insight | 30% |
| Link to course concepts | 25% |
| Professionalism and clarity | 15% |
| Visual documentation (where required) | 10% |

**Appendix S4. COM353 Post-Block Student Feedback Questionnaire (42 Items)**

The instrument below is the post-block student feedback questionnaire administered to the cohort. It was adapted from the Dundee Ready Education Environment Measure (DREEM) and the Groningen Reflection Ability Scale (GRAS) and supplemented with items developed for domains central to COM353 but not fully captured by those tools (uncertainty tolerance in primary-care settings, experiential learning, and systems thinking). All 42 scaled items used a five-point Likert scale: 1 = Strongly Disagree, 2 = Disagree, 3 = Neutral, 4 = Agree, 5 = Strongly Agree. The survey closed with a global block rating (item 43) and one open-ended item (item 44). Items are reproduced verbatim as administered and are grouped by the seven a priori domains used in the analysis.

**Domain 1. Learning Environment and Teaching Quality (items 1 to 7)**

1. The COM353 block promoted active learning rather than passive memorization.

2. The learning objectives of COM353 were clear and consistently addressed.

3. Teaching sessions were well organized and logically structured.

4. Faculty encouraged questions, discussion, and critical thinking.

5. I felt comfortable expressing uncertainty during teaching sessions.

6. The learning environment was respectful and supportive.

7. The balance between lectures, discussions, and experiential activities was appropriate.

**Domain 2. Family Medicine and Primary Health Care Competencies (items 8 to 14)**

8. COM353 improved my understanding of the core principles of Family Medicine.

9. I better understand the role of Primary Health Care in the healthcare system.

10. The block enhanced my understanding of continuity of care.

11. I gained insight into comprehensive care across age groups and conditions.

12. I better understand the importance of preventive care and health promotion.

13. The block highlighted the role of family physicians as first-contact clinicians.

14. I developed a clearer understanding of the scope of practice of family physicians.

**Domain 3. Experiential Learning and Clinical Exposure (items 15 to 20)**

15. The PHC visits were well integrated into the COM353 block.

16. PHC visits helped me apply theoretical knowledge to real clinical settings.

17. I observed patient-centered care during PHC visits.

18. PHC exposure improved my understanding of long-term patient management.

19. I observed how family physicians address multiple concerns in a single visit.

20. PHC visits helped me understand care beyond diagnosis and prescriptions.

**Domain 4. Clinical Courage and Uncertainty Tolerance (items 21 to 26)**

21. COM353 helped me become more comfortable managing clinical uncertainty.

22. I developed confidence in engaging with patients despite incomplete information.

23. The block encouraged clinical courage, including speaking up when unsure.

24. I am more comfortable acknowledging limitations in my knowledge.

25. I learned to tolerate ambiguity in clinical decision-making.

26. COM353 emphasized thoughtful reasoning rather than defensive medicine.

**Domain 5. Reflection and Professional Identity Formation (items 27 to 32)**

27. Reflective activities helped me critically analyze my clinical experiences.

28. Writing reflections helped me link experiences to professional values.

29. Reflection increased my awareness of my assumptions and biases.

30. Reflection helped me integrate ethical, emotional, and clinical perspectives.

31. I am more comfortable reflecting on challenging or uncomfortable encounters.

32. The reflective component contributed to my development as a future physician.

**Domain 6. Systems Thinking and Community Orientation (items 33 to 37)**

33. COM353 improved my understanding of healthcare as a system.

34. I gained insight into referral pathways and coordination of care.

35. The block emphasized collaboration with other healthcare professionals.

36. I better understand the physician's role in community-oriented care.

37. The block highlighted issues of equity, access, and social determinants of health.

**Domain 7. Overall Impact and Readiness (items 38 to 42)**

38. COM353 influenced how I view my future clinical practice.

39. The block prepared me for future clinical rotations.

40. COM353 increased my interest in Primary Care and/or Family Medicine.

41. The skills developed in COM353 are relevant regardless of specialty choice.

42. Overall, COM353 was a valuable and effective block.

**Closing items**

43. Overall, how would you rate the COM353 block? (global rating)

44. Which aspect of the Primary Health Care experience had the greatest impact on your learning, and why? (open-ended)

**S4.1 Per-item corrected item-total correlations (first delivered cohort, N = 96)**

The table below reports the corrected item-total correlation (CITC) for each of the 42 items in the post-block survey. Item numbering corresponds to the instrument reproduced in Appendix S4 above. Domain labels follow the seven a priori domains: D1, Learning Environment and Teaching Quality (items 1 to 7); D2, Family Medicine and PHC Competencies (8 to 14); D3, Experiential Learning and Clinical Exposure (15 to 20); D4, Clinical Courage and Uncertainty Tolerance (21 to 26); D5, Reflection and Professional Identity Formation (27 to 32); D6, Systems Thinking and Community Orientation (33 to 37); D7, Overall Impact and Readiness (38 to 42). CITC values range from 0.675 to 0.900 (mean 0.809, median 0.809), with 41 of 42 items (98%) at 0.70 or higher and 23 items (55%) at 0.80 or higher. The mean inter-item correlation across all 861 unique item pairs is 0.664. These values, together with the overall Cronbach's alpha of 0.988, indicate that the items are largely indexing one broad construct rather than seven empirically distinct domains; the domain-level regression coefficients in the main paper Table 1 should therefore be treated as exploratory. See main paper Section 4.4 (final paragraph) and Section 6 for full interpretation.

| **Item** | **Domain** | **CITC** | **Interpretation** |
| --- | --- | --- | --- |
| 1 | D1 | 0.744 | High |
| 2 | D1 | 0.761 | High |
| 3 | D1 | 0.719 | High |
| 4 | D1 | 0.870 | Very high |
| 5 | D1 | 0.759 | High |
| 6 | D1 | 0.857 | Very high |
| 7 | D1 | 0.809 | Very high |
| 8 | D2 | 0.849 | Very high |
| 9 | D2 | 0.807 | Very high |
| 10 | D2 | 0.815 | Very high |
| 11 | D2 | 0.820 | Very high |
| 12 | D2 | 0.893 | Very high |
| 13 | D2 | 0.798 | High |
| 14 | D2 | 0.809 | Very high |
| 15 | D3 | 0.711 | High |
| 16 | D3 | 0.675 | Moderate |
| 17 | D3 | 0.726 | High |
| 18 | D3 | 0.764 | High |
| 19 | D3 | 0.730 | High |
| 20 | D3 | 0.721 | High |
| 21 | D4 | 0.763 | High |
| 22 | D4 | 0.788 | High |
| 23 | D4 | 0.836 | Very high |
| 24 | D4 | 0.869 | Very high |
| 25 | D4 | 0.876 | Very high |
| 26 | D4 | 0.897 | Very high |
| 27 | D5 | 0.747 | High |
| 28 | D5 | 0.777 | High |
| 29 | D5 | 0.758 | High |
| 30 | D5 | 0.774 | High |
| 31 | D5 | 0.792 | High |
| 32 | D5 | 0.765 | High |
| 33 | D6 | 0.900 | Very high |
| 34 | D6 | 0.849 | Very high |
| 35 | D6 | 0.869 | Very high |
| 36 | D6 | 0.874 | Very high |
| 37 | D6 | 0.886 | Very high |
| 38 | D7 | 0.853 | Very high |
| 39 | D7 | 0.896 | Very high |
| 40 | D7 | 0.844 | Very high |
| 41 | D7 | 0.884 | Very high |
| 42 | D7 | 0.859 | Very high |

**Reference cited in this Supplementary Material**

Phillips, A. C., Lewis, L. K., McEvoy, M. P., Galipeau, J., Glasziou, P., Moher, D., Tilson, J. K., and Williams, M. T. (2016). Development and validation of the guideline for reporting evidence-based practice educational interventions and teaching (GREET). BMC Med. Educ. 16, 237. doi: 10.1186/s12909-016-0759-1
